# Patterns of Health Services and Medicine Utilisation by First‐Generation Pakistani Immigrants in New Zealand

**DOI:** 10.1111/hex.70169

**Published:** 2025-02-02

**Authors:** Noor A. Mann, Zain A. Khan, Saima Asghar, Afshan Rani, Nadia Hussain, Sumera S. Akhtar, Susan Heydon, Mudassir Anwar

**Affiliations:** ^1^ School of Pharmacy University of Otago Dunedin Otago New Zealand; ^2^ Department of Pharmacy Practice The Islamia University of Bahawalpur Bahawalpur Punjab Pakistan; ^3^ College of Education University of Otago Dunedin Otago New Zealand; ^4^ Department of Pharmaceutical Sciences, College of Pharmacy Al Ain University Al Ain Abu Dhabi United Arab Emirates; ^5^ Centre for International Health University of Otago Dunedin Otago New Zealand

**Keywords:** health services, immigrants, New Zealand, patient satisfaction, self‐medication

## Abstract

**Introduction:**

The health‐seeking patterns of the increasing Pakistani migrant population in New Zealand (NZ) is a subject of limited research in current literature. Therefore, the study aimed to investigate and understand the patterns of health services utilisation and medicine usage among first‐generation Pakistani immigrants in NZ.

**Methods:**

Convenience and snowball sampling using social media platforms were used to conduct eleven semi‐structured interviews consisting of two participants per interview. The interviews were audio‐taped and transcribed verbatim. Data were analysed iteratively using an inductive thematic approach.

**Results:**

Self‐medication emerged as a common practice by the majority of the participants and this practice stemmed from personal experiences, existing knowledge, and personal and cultural beliefs. However, participants had limited knowledge of the NZ health system and community pharmacy services and this led to conflicting expectations and outcomes. These factors serve as barriers to healthcare access for Pakistani immigrants and can result in adverse health outcomes and higher rates of dissatisfaction.

**Conclusion:**

This study highlights the healthcare challenges and behaviours of Pakistani immigrants in NZ, emphasising their reliance on traditional remedies and self‐medication. While appreciating the quality of NZ's healthcare, participants faced barriers like cost and lack of awareness of pharmacy services. The findings call for culturally tailored strategies to improve accessibility and healthcare experiences for immigrants.

**Patient or Public Contribution:**

Two individuals from the Pakistani community, not part of the study, contributed to the design by offering feedback and pilot testing the interview guide. Based on their input, adjustments were made to enhance the clarity of the questions from a patient's or public viewpoint. No new questions were proposed as a result of this feedback.

## 1 Introduction

In the current climate of globalisation, New Zealand (NZ) is witnessing a burgeoning migrant population that has escalating health needs. This poses a challenge to the health system as the migrant populations bring unique health needs, including different cultural practices, language barriers, and varying levels of understanding of the healthcare system, which can complicate the delivery of care. Various factors drive the migration to a new country including the possibility of improving employment opportunities, a refuge from political unrest, educational opportunities, health benefits and an overall favourable environment [1]. Migration does not come without its issues and those who take this step face several difficulties in navigating healthcare systems due to the unfamiliarity of the social, structural, and cultural practices unique to their new place of residence [2]. This creates barriers to access, frequently leading to health disparities that are manifested through poor health outcomes [3].

Pakistan, the fifth most populous country in the world, comprises several primary ethnic groups including the main ones such as Punjabis, Pashtuns, Sindhis, and Balochis. The healthcare system in Pakistan is divided into a predominantly underfunded or unfunded public healthcare system and an exorbitantly expensive private healthcare system. Despite the cost associated, up to 79% of the Pakistani population opt for private healthcare services in Pakistan due to higher patient satisfaction [4]. The 2018 NZ census population data indicated there are 6135 Pakistanis residing in the country with the majority (75.9%) having been born overseas and migrated at various points in their lives [5]. A significant proportion falls within the 20 – 69 age range, with 57% of male and 53.2% of female Pakistani migrants belonging to this age demographic [5].

Health literacy is defined as one's ability to access, understand, appraise, and apply health information to make judgements and take decisions in everyday life concerning healthcare, disease prevention, and health promotion [6]. This proficiency allows individuals to be empowered enough to make well‐informed and effective medical decisions [6]. Recognised globally by healthcare professionals and researchers, health literacy is an important determinant of the health and health‐seeking behaviour of immigrants [7]. Health literacy levels are independent of the educational background of any population [8]. At least 44.1% of Pakistanis in NZ possess advanced qualifications with at least a bachelor's degree and are actively employed within a variety of professional sectors [5]. These statistics underscore the high qualification of Pakistani immigrants in NZ; however, considering the contrasting health systems of the two countries, there may be gaps in their knowledge and awareness of the healthcare system. Consequently, these gaps could potentially contribute to challenges including limited access, delays in treatment, and areas of miscommunication between the healthcare providers and patients [8].

One of the examples of how the healthcare systems in the two countries vary is the role of the pharmacists. In Pakistan, over half of registered pharmacists gravitate towards employment within the pharmaceutical industry, while the remaining percentage engages in diverse professional settings, encompassing community and hospital pharmacies, pharmaceutical marketing, and regulatory affairs. Notably, the clinical involvement of pharmacists in these roles is either restricted or absent altogether [9]. Conversely, in NZ, pharmacists play a multifaceted role within the healthcare landscape. Beyond dispensing medications, they are integral members of healthcare teams, providing medication counselling, management of chronic conditions, and vaccination services. Pharmacists also engage in medication reviews, ensuring the safe and effective use of medications, and contribute to public health initiatives such as smoking cessation programs and health promotion campaigns. Additionally, they play a pivotal role in pharmacovigilance, reporting adverse drug reactions to safeguard patient safety. The lack of recognition in the healthcare system has led to the under‐utilisation of pharmacists which has consequently limited the interaction between pharmacists and patients in Pakistan [10]. As a consequence, migrants from Pakistan may face challenges in drawing parallels between the healthcare systems of the two countries. The experience of migrants in NZ may not be that pharmacists are important in addressing community health issues. This is especially true if the migrants are from countries with significantly different healthcare systems. This potential misalignment could pose challenges in obtaining timely healthcare in NZ, where pharmacists play a significantly larger role in addressing and alleviating healthcare concerns of the community.

Our study aimed to explore the healthcare‐seeking behaviours, awareness, and experiences of Pakistani immigrants in NZ. The study also aims to identify barriers and opportunities for improving healthcare delivery and support for this community through culturally tailored strategies. We primarily focused on the experiences of first‐generation Pakistani immigrants and their families within the NZ healthcare system. These individuals were born in Pakistan and later migrated to NZ. This population has specific cultural practices and beliefs about healthcare, as well as potential language barriers, which can affect how they navigate the healthcare system. By understanding these unique challenges, we aim to inform more culturally sensitive healthcare practices that better address the needs of this growing population in NZ.

## 2 Methods

### 2.1 Study Design

This qualitative study used semi‐structured household interviews, guided by a topic guide with prompts (Supplementary Material), as the primary method of data collection to analyse the patterns of health services and medicine utilisation among Pakistani immigrants in NZ. The topic guide was designed to explore a variety of issues related to healthcare utilisation. The purpose of the open‐ended and closed‐ended questions was to capture patterns of behaviour. A copy of the topic guide is included as supplementary material to provide a clear understanding of the interview framework.

### 2.2 Participant Recruitment

Participants were recruited through a combination of social networking, utilising a snowball technique, and disseminating the study flyer on multiple social media platforms such as Facebook and WhatsApp. The participants were recruited from various locations around the North and South Islands of NZ. The eligibility criteria to participate in this study included individuals who were first‐generational Pakistanis, had legal residence status in NZ, were at least 18 years old, and willingly consented to take part in the interviews.

### 2.3 Data Collection and Analysis

The lead researcher, MA, devised a topic guide with prompts (Supplementary Material) as a guiding framework for the in‐person interviews. Additional questions that were tailored to the participants were also asked to ensure sufficient information was gathered during the interview. The questions were aimed to shed light on the participants' perspectives and understanding of the NZ health system. Examples of the questions included “What is your general impression of the NZ health system?,” “What difficulties/barriers do you experience when accessing healthcare in NZ?,” and “Are you aware of the services community pharmacy have to offer?.” We focused on the collective perspectives of households as a unit, considering the shared decision‐making process around health‐seeking behaviours. Each household interview consisted of two participants who were husband and wife. Data saturation was monitored throughout the interview process in the study, and we determined it had been reached when no new themes or insights emerged from the data. This was achieved after 11 interviews [11]. The interview time duration was between 45 and 75 min. Given that Urdu and English are the two official languages of Pakistan, with Urdu being more widely spoken and understood by the majority, the lead researcher, MA, used Urdu to conduct all interviews as well as audio‐recorded them with participant consent. Subsequently, all interviews were transcribed verbatim and translated into English by SSA. Field notes were maintained to document the interviewer's interpretations and perceptions during each session. Before coding the data, the researchers familiarised themselves with all the interviews to develop the themes, sub‐themes, and categories. After the first few interviews, initial coding began to allow for further refinement and adjustment of the coding framework. Thematic saturation was determined when no new themes emerged during the subsequent interviews. Additionally, field notes documented the interviewer's interpretations and perceptions, which were incorporated into the analysis to enhance understanding of the data. The thematic analysis was conducted according to the Braun and Clarke guidelines and the N‐vivo software was used for data analysis [12]. To ensure analytical rigour, the research team practised reflexivity throughout the study, with team members regularly reflecting on their assumptions and how these might influence the analysis.

## 3 Results

The mean age of the participants in this study was 40.9 years, with all households having older husbands than wives, and there was a 1:1 ratio of males and females. Most participants were employed and most commonly belonged to the engineering profession while most of the women were housewives. The participants in this study were located in Dunedin, Wellington, Auckland, and New Plymouth. The demographic details of the participants are presented in Table 1.

**Table 1:** Demographic characteristics.

Interview	Age(s)	Relationship	Occupation
1	65	Husband	Engineer
	55	Wife	Housewife
2	43	Husband	IT specialist
	38	Wife	Housewife
3	42	Husband	Engineer
	36	Wife	Housewife
4	43	Husband	Engineer
	37	Wife	Housewife
5	35	Husband	Engineer
	31	Wife	Commercial analyst
6	50	Husband	Criminal Barrister
	46	Wife	Housewife
7	44	Husband	Auto technician
	39	Wife	Beautician
8	37	Husband	Engineer
	32	Wife	Housewife
9	38	Husband	Data scientist
	33	Wife	Housewife
10	41	Husband	Engineer
	38	Wife	Student
11	41	Husband	Investment manager
	37	Wife	Housewife

Four core themes were inductively identified: healthcare‐seeking behaviour; cognisance of the NZ healthcare system; Aspects of healthcare quality that are important to Pakistani immigrants; and Difficulties or barriers while accessing healthcare in NZ. Illustrative quotes are provided in the text, with themes summarised in Table 2.

**Table 2:** Summary of themes.

Themes	Subthemes
Healthcare‐seeking behaviour of Pakistani immigrants in NZ	Use of home remedies and traditional medicines in minor illnesses Sel‐medication Visit to a GP Bringing medicines from Pakistan for use in NZ
Cognisance of the NZ healthcare system	Views about healthcare delivery system Sources of information regarding the healthcare system
Aspects of healthcare quality that are important to Pakistani immigrants	Key Expectations and Preferences for GP Characteristics Community pharmacy services
Difficulties/barriers while accessing healthcare in NZ	Cost of GP visit, health insurance, and dental care Referral services

### 3.1 Healthcare‐Seeking Behaviour of Pakistani Immigrants in NZ

Participants' healthcare‐seeking behaviour revealed a reliance on both traditional and conventional medical practices, highlighting how cultural values and personal health beliefs influence decisions about when and where to seek care.

#### 3.1.1 Use of Home Remedies and Traditional Medicines in Minor Illnesses

The participants of this study displayed a reliance on using home remedies for minor ailments. Home remedies were the preferred initial treatment strategy for children suffering minor illnesses in the confines of their homes. In instances where home remedies were not treating the condition adequately, a visit to healthcare providers such as general practitioners (GPs) was generally the next preferred option. Participants considered herbal remedies, such as Joshanda (a herbal mixture marketed to treat cold and flu symptoms), as separate from home remedies that used ingredients such as garlic, ginger, honey, and fennel seeds.

My children are also in the habit of taking home remedies as I have been giving them from childhood, such as Joshandha. Whenever they get sick, they ask for home remedies, and they say can we have some home remedies before medicines?

[Interview 1: Wife]

I take home remedies first. Like if there is a headache, fever, or flu, I give a phone call to my mother in Pakistan and tell her the symptoms. For example, I tell my mother that I have tummy aches so what should I do? She would say, give fennel. Mother remedies always work. Then, if still not recovering or it is viral or something I call a GP.

[Interview 6: Wife]

#### 3.1.2 Self‐Medication With Conventional Medicines

The majority of the parents reported reliance on self‐medication, particularly when experiencing the early symptoms of common conditions such as cough, colds, and general aches and pains. The most commonly used medications included paracetamol and antihistamines to treat common ailments. Interestingly, some parents expressed their ability to assess the severity of their symptoms and whether self‐medication would be sufficient or a visit to the GP was warranted.

We only use antiallergics and paracetamol for fever. We both take blood pressure medicines. So we avoid taking any other medications.

[Interview 4: Wife]

Yes, we do use home remedies as our children do not like to take conventional medicines. The only medicine we give them, that too with difficulty, is paracetamol.

[Interview 5: Wife]

We see what has happened. Most of the time paracetamol works. If the condition persists for more than a day, we will go to a GP.

[Interview 7: Husband]

We self‐cure and self‐medicate ourselves. We use paracetamol as the first resort.

[Interview 8: Husband]

#### 3.1.3 Visit to a General Practitioner (GP)

A significant proportion of the study participants shared their inclination to visit the GP when their child was sick. The preference was clearly for their children, whereas for themselves, they preferred to avoid the cost and the long waiting times involved in consulting a GP. Depending on the condition's severity, most parents choose to use herbal remedies and over‐the‐counter (OTC) preparations first before visiting GPs.

I even give children tea and paracetamol. If they don't get better until the third day, then I will take them to the GP.

[Interview 9: Wife]

Of course, in Pakistan, we diagnose ourselves and bring medicines for ourselves, but here in NZ, we do not do this, e.g., if someone has a little bit of cough, you get cough syrup from the supermarket. But if the condition is severe then we directly go to a GP.

[Interview 10: Husband]

For children, we know that the younger ones will not be able to recover. Then we call the medical centre. Then the nurse calls back and she asks about the symptoms, and then if she thinks there is a need, she books an appointment with the GP the same day. The second alternative is taking them to the hospital emergency if there is a holiday the next day. You have to wait there, but they see them.

[Interview 11: Husband]

It depends; for example, if a child has a fever, then we do self‐remedy, we give paracetamol at home. If he is not recovering then we take them immediately to the GP. With children, you have more fear factor. Children cannot tell you exactly what symptoms they have.

[Interview 2: Husband]

#### 3.1.4 Bringing Medicines From Pakistan for Use in NZ

A common pattern and behaviour observed in the majority of Pakistani immigrants was bringing various medicines from Pakistan for casual use. The most popular types of medicines brought from Pakistan included antibiotics and painkillers. Most immigrants felt the need to resort to sourcing such medicines from Pakistan, as they considered them to be difficult to access or, in some cases, inaccessible in NZ. The participants expressed a perceived necessity of antibiotics and these expectations were not being met in the NZ healthcare system, compared to the availability and ease of access in Pakistan. This is due to NZ having a more regulated approach in terms of antibiotic prescription and sale to prevent inappropriate utilisation.

You cannot quickly get antibiotics from here. Even if you ask the doctor, they will not give you antibiotics immediately.

[Interview 2: Wife]

Sometimes, we bring some medicines. We sometimes bring medicines for flu, including antibiotics. We also bring Panadol CF® (paracetamol for cold and flu).

[Interview 9: Husband]

Yes, if any friend is coming, usually painkillers, some antibiotics, and over‐the‐counter medicines. The idea here is that you will get better on your own. As we are in the habit of using antibiotics and if a doctor does not prescribe them, we feel they are not a good doctor and have wasted our time and money. Initially, we feel the need, but as you spend time here, you get used to the system and now we never ask for antibiotics from Pakistan.

[Interview 11: Husband]

### 3.2 Cognisance of the NZ Healthcare System

This theme explores participants' awareness and understanding of the NZ healthcare system, highlighting their positive perceptions of its delivery, the respect and professionalism of healthcare staff, and the comprehensive treatment options available. Additionally, the theme touches on the sources of information that helped participants navigate the system.

#### 3.2.1 Views About Healthcare Delivery System

Based on their collective experience, the participants had a positive view of NZ's healthcare system and described it as effective and efficient in treating healthcare concerns. Participants noted that the coverage and treatment were comprehensive. Most participants also expressed favourable opinions concerning healthcare staff, emphasising that they found them to be respectful and overall satisfactory. Several participants understood the rationale of limiting antibiotic usage and that further investigations were done before antibiotics were provided, unlike in Pakistan where antibiotics were easily prescribed. Some of the participants were aware of services related to mental health including the free helpline for depression and mental stress. This service considered unconventional and not readily available in Pakistan, has a cultural stigma attached to it, so participants found that the NZ healthcare system offering this service was of significant benefit.

I think the way doctors behave is excellent. They treat you really well. The doctors are empathetic.

The staff is caring, whenever we go to the hospital. They take care of hygiene a lot. Medicine quality is also excellent.

[Interview 5: Husband, Wife]

They do not let you wait and give you protocol. You know in Pakistan, how they treat. Here the doctor comes out of his office to receive you with a greeting and respect, which is good. They also give you a follow‐up call to see how you are doing.

[Interview 3: Wife]

I like that rather than looking at symptoms they go for the investigation. If you tell them my throat is infected and I have pain in my throat, they will not give you antibiotics. They will do a test and investigate further. I like this.

[Interview 3: Husband]

#### 3.2.2 Sources of Information Regarding the Healthcare System

The major sources of information regarding NZ's healthcare system were friends, colleagues, and community members. The focus was predominantly on how to access a doctor and the process of registering at a medical practice. These connections enabled the new immigrants to navigate through NZ's healthcare system. These sources of information were predominantly based on the individual's personal experience or their exposure to representatives of the local government healthcare system.

My friends told me that you first go to the nearby clinic and get yourself registered with a GP once you arrive. So I went and got registered there.

[Interview 3: Husband]

Before going to the GP, kindergarten people guided us a lot, for example, about Plunket and immunisation. They gave us a lot of information.

[Interview 6: Husband]

From friends and colleagues etc. Then we got the resident pack that also includes healthcare information. The first piece of information that I found crucial was to register with a GP was very important. So we accessed the GP and did registration. As our children were small, we talked to the GP about childhood vaccination. So most of the information was from friends and family.

[Interview 8: Husband]

### 3.3 Aspects of Healthcare Quality That Are Important to Pakistani Immigrants

This theme examines the key factors that influence Pakistani immigrants' perceptions of healthcare quality in NZ, focusing on their expectations for GPs and community pharmacy services.

#### 3.3.1 Key Expectations and Preferences for GP Characteristics

Immigrant Pakistanis in NZ displayed similar patterns highlighting common themes when describing their expectations and preferences of an ideal GP. Their experiences consequently impact their perception regarding the quality of the healthcare provided. Overall, most participants stated that they expect GPs to listen actively and provide a thorough assessment of their health each time. The participants also emphasised that GPs should be able to derive an accurate diagnosis from the initial appointment to minimise delays in seeking prompt treatment. The participants in this study generally preferred younger GPs who graduated from NZ, as these GPs were perceived to have the most updated knowledge by the interviewed group. Participants also expressed that GPs should be mentally and physically fit to work and provide optimal healthcare to patients. The distance from a patient's residence to the GP clinic was a major factor in the level of satisfaction for patients. GP clinics that were in close proximity were generally favoured due to their ease of access. Additionally, cost was a major driving factor for most patients. The cost determined whether they would choose to sign up for a specific GP as the cheaper GP was usually the most preferred option.

He checks thoroughly. He takes a full history and gives proper time. He should check adequately, listen carefully, ask in detail, and understand the condition.

[Interview 1: Husband and wife]

The reason for this is if you go to the GP, the first thing is what he diagnoses. So from a diagnosing point of view, they are very weak. First time, they will just give you paracetamol. You go second time, then third and fourth time. After the third and fourth time, they will realise that something is seriously wrong; then they will diagnose properly or refer you to a specialist.

[Interview 2: Husband]

The fresh graduates nowadays are good, they have different courses for new graduates from the older ones. Now the new GP is learning through a different but advanced process that also involves research.

[Interview 10: Husband]

#### 3.3.2 Community Pharmacy Services

A few interviewees were slightly more familiar with the services pharmacies across NZ provided than many others. Most of them knew the role of community pharmacies in providing OTC medicines without a prescription. Most participants who recollected a helpful experience with a pharmacist praised their ability to take history, diagnose, and treat minor conditions. However, a large proportion of participants were unaware of extra services provided at pharmacies such as blood pressure monitoring, home delivery of medicines, blood glucose monitoring, and vaccinations. Hence, their first point of contact for their health needs leaned towards their GPs instead. Furthermore, one interviewee suggested that conflicts in opinions between GPs and pharmacists should be eliminated as it can lead to confusion and heighten patient frustration.

They can give cough syrup, paracetamol, tablets, Vicks®, bandaging, and anything without a prescription.

[Interview 1: Husband]

I do not know any as I told you I went to buy some medicines and she asked me to get a prescription from a GP. I told her that it was a holiday, how can I find a GP. Then she asked me to wait, and when the pharmacist came, she took history and then gave me medicine but after taking notes. I think she may have to put the details in our medical record.

[Interview 3: Husband]

We know medicine dispensing, but we do not know medicine management service, vaccination service, gout, UTI, pharmacy clinic, blood pressure, blood glucose, weight management, smoking cessation, addiction, Pharmacist prescribing, bone density etc.

[Interview 7: Husband and wife]

But then there was a conflict between pharmacists and doctors from fracture clinics. The pharmacist said that you could use this sling and the fracture clinic said no, you could not use it as your hand will not move properly.

[Interview 9: Husband]

### 3.4 Difficulties/Barriers While Accessing Healthcare in NZ

This theme explores the challenges faced by Pakistani immigrants in New Zealand when trying to access healthcare, focusing on financial barriers, delays in referral services, and limited access to specialists.

#### 3.4.1 Cost of GP Visit, Health Insurance, and Dental Care

The majority of the study participants, as most NZers, did not have any form of healthcare insurance unless their places of employment provided for it. The factors contributing to their lack of healthcare insurance included the perception of it being an additional and unnecessary financial burden since medical treatment is subsidised for all NZ residents. Participants also utilised Accident Compensation Corporation (ACC) to cover their health‐related costs related to accidents or sports injuries. The government‐funded healthcare system of NZ mitigated the necessity of healthcare insurance, which distinguishes it from the healthcare system of many other countries.

We never bought insurance for ourselves. I do have insurance from my company. The main reason why we wouldn't buy it ourselves is because we do not have any chronic disease yet, and hence we do not have to see a GP frequently.

[Interview 2: Husband]

No, The reason for this is, that I only had insurance during student life as it was compulsory. But now we don't think there is a need since we do not have any illness. If you go to a hospital emergency, they will treat you somehow, so do not feel the need for insurance.

[Interview 5: Husband]

Many participants reported that the cost of GP fees and dental services was a barrier to accessing the services especially if they did not have health insurance.

I feel like it is expensive. So finance is a barrier. If you do not have insurance, then it is a big hurdle, and you have to think that 50 to 60 dollars is a GP fee, and you never know how long the conditions prolong, so cost is a barrier. Dental services are very expensive. If you have no insurance, then it is a big hurdle.

[Interview 9: Husband]

#### 3.4.2 Referral Services

Referral services have been an area of challenge for Pakistani immigrants and the process of being referred by the GP to the relevant specialised was described as being time‐consuming. This delay in receiving prompt assessment by medical professionals was believed to be a major contributor to the deterioration of the participants' health. The participants were accustomed to the far easier access to a medical specialist for their health needs in Pakistan, while in NZ, GPs are the most accessible medical professionals in the community and most patients are directed to them instead of a specialist. This disparity between the two healthcare systems leads to unmet expectations and introduces additional barriers for Pakistani immigrants to accessing healthcare services in NZ.

The first thing is that it wastes time, and physically you get deteriorated. After two to 3 months, you reach a specialist, then the specialist starts the process and it takes two to 3 months to finalise it. So if you have to rectify a disease it will at least take 5 to 6 months.

[Interview 2: Husband]

They refer you to the GP. If it's an emergency, anything can happen to that person. Until GP refers you to the specialist, anything can happen to that person.

[Interview 7: Wife]

As we are used to taking children to the paediatrics and for every problem, we have to go to that specialist. Here, we cannot go to the specialist until the GP recommends you. Here, we have to go to the GP. Maybe they want us to take as fewer medications as possible.

[Interview 4: Wife]

## 4 Discussion

This study examined the patterns of health services and medicine usage amongst first‐generation Pakistani immigrants in NZ. The participants predominantly had a positive perspective on NZ's healthcare system, the influence of their past experiences shaped their expectations and level of satisfaction with their current healthcare providers and system. These experiences were reflective of participants' behaviour and understanding of the NZ health system which also revealed barriers that hampered the extensive utilisation of healthcare services.

A recurrent trend that this study identified was the use of self‐medication for minor ailments, preceding any decision to consult a healthcare professional. This included using OTC medications, herbal and traditional medicines, home remedies, and medicines brought over from Pakistan. The types of medicines that were often brought from Pakistan included pain relief medications and antibiotics. This was in line with the study by Akhtar et al. where the list of medications most often brought from Pakistan included antiemetics, antibiotics, antidiarrhoeals, laxatives, cough, flu, and cold medications at antipyretics, anti‐allergic medications, and even topical steroid creams [13]. The study also highlighted that choosing the medicine, dosage, and interval was most often decided by the mothers in the household by consulting family and friends, information online and in the leaflets of the medications [13]. This behaviour stems from differences in the healthcare systems of Pakistan and NZ and could lead to misalignment of expectations. The accessibility and over‐prescription of antibiotics in Pakistan contribute to the anticipation of frequent antibiotic use among Pakistani migrants in NZ, quite often leading to dissatisfaction with the healthcare services [14]. Compared to Pakistan, NZ healthcare has emphasised antimicrobial stewardship resulting in a significant reduction in antibiotic dispensing from 2015 onwards [15]. Studies such as those by Lescure et al. have highlighted the trend that GPs often experience pressure from immigrant patients to prescribe antibiotics and even face issues in communicating this in a culturally sensitive way [16].

Another contributor to self‐medication is the inaccessibility of NZ prescribers due to extended wait times and the associated costs. Subsequently, participants often exhaust all known treatments at home based on their personal knowledge and experiences before resorting to visiting a healthcare professional. In Pakistan, the common practice of self‐medication is partly due to the ease of access to obtaining medicines without a prescription [17].

In cases involving children, the study participants did not delay in seeking consultations with their GPs and starting conventional treatment. Parents in this study demonstrated protective behaviour towards their children, prioritising professional treatment to prevent any exacerbations. This trend may be attributed to the free medical services for children in NZ. The minimal cost associated with paediatric health serves as a facilitator, enhancing access and utilisation of such medical services provided in NZ [18].

In contrast, the study by Akhtar et al. indicated that parents often treated their children for colds, fever, or diarrhoea for up to 3 days at home before seeing further professional healthcare treatment. Immediate medical attention was reserved for serious injuries instead. The research found that mothers, in particular, were reluctant to visit the GP unless a serious injury was involved and the hesitancy was because the GP would most likely not provide them with adequate medications to treat the illness and that often their opinions were dismissed [19].

Our study also highlighted the active involvement of the wives and mothers in providing care for the unwell members of their households and this recurrent theme was found amongst the majority of the participants and was consistent with the earlier findings by another researcher [13]. It was evident that wives and mothers had a notably proactive role in caring for sick family members. Most of the participant responses suggested the role taken by the females included assessment or triage and treatment of the unwell family member as well as preparing and administering the home remedies. The role encompassed the identification and management of familiar symptoms and illnesses using a combination of Western medicines, traditional medicines, and/or home remedies that had previously shown effectiveness in alleviating the symptoms [20]. The practice of self‐diagnosis in Pakistani households was characterised by the common storage of medicine pre‐emptively for future use. Research indicates that amongst Pakistani households the most commonly stored medications include analgesics, antibiotics, cough and cold medicines, anti‐diarrhoeal, and antiemetic agents [21]. This practice, coupled with the ability to purchase prescription medicines without a prescription in Pakistan, allows individuals to readily access medicines for prompt symptomatic relief [21]. While this practice persists post‐migration, there is a disparity in the expectations when dealing with the NZ healthcare system. In contrast to Pakistan, medications are rigorously regulated in NZ and are not easily obtained without the involvement of a healthcare provider. Although this practice has followed through post‐migration, there is a mismatch in expectations from the NZ health system. These regulations are vital to reduce the risk of medicine misuse but can inadvertently limit access. Additionally, there is evidence suggesting that self‐diagnosis and self‐treatment at home are associated with the risk of administering inappropriate medicines and delaying treatment which can result in overall poor health outcomes [22, 23].

In Pakistan, people are accustomed to visiting hospitals to consult with doctors or specialists for any medical concerns. These expectations are carried onwards by Pakistani migrants in NZ and the longer wait time associated with NZ's referral process is perceived as a barrier reducing access to specialist care. Migrants also had the expectation of a comprehensive diagnosis during the first meeting with their treating doctor, though this is not feasible in every case and many patients require further investigations. These unmet expectations contribute to participants forming a perception that their health was not being adequately prioritised. Additionally, many Pakistani immigrants were not utilising other health services, placing an increased burden on general practitioners (GPs). The ongoing GP crisis in NZ further adds to the challenge of getting prompt appointments [24]. The higher costs and limited services associated with private hospitals in NZ prevent utilisation leaving patients to wait for public health services, often involving longer waiting periods [25]. This is in contrast to the situation in Pakistan where the private healthcare sector is commonly utilised and associated with significantly shorter wait times, as well as being able to cope with the growing healthcare needs of the population [26, 27].

A significant proportion of the study participants had a lack of awareness when questioned about their knowledge of services provided by NZ's community pharmacies. This is in line with the study by Abdul Aziz et al. where pharmacists provide many professional services without remuneration and yet remain underutilized by the public [28]. This seems to be a recurring theme in Pakistani immigrants moving to high‐income countries such as NZ [29]. Consequently, GPs remain their primary point of contact for various health concerns irrespective of their severity. Due to the substantial differences in the structure, function, and overall role of NZ community pharmacies when compared to Pakistan, migrant Pakistanis face challenges in the appropriate usage of NZ's well‐developed pharmacy services. The role of community pharmacists has evolved beyond that of dispensing, allowing a more active role in providing healthcare. In NZ, pharmacists now provide several services that are similar to GPs, including reviewing medicine use, chronic or long‐term condition services, selected oral contraceptives, blood pressure monitoring, anticoagulant monitoring services, and vaccinations [30]. These services have been expanded to allow community pharmacists to help cope with the high influx of complicated medical conditions coupled with GP shortages [31]. However, our study revealed that the Pakistani migrants were predominantly unaware of these services and this could be explained by their sources of information focusing on GPs as the initial point of care. There is a knowledge gap regarding other health services, as their information sources are limited to family and close friends within the same community. Consequently, the expectations of Pakistani migrants concerning healthcare providers are not met and this contributes to their ineffective utilisation of NZ's health services. This pattern can be justified by the unfamiliarity with and dissimilarity between the different health systems in both countries, further accentuating the observed pattern of self‐diagnosis and self‐medication in this particular population.

### 4.1 Study Limitations

This research did not analyse the regional differences which may directly or indirectly influence the health‐seeking behaviours observed in the Pakistani migrant population of NZ. There are six main regions in Pakistan with distinct cultural norms that may impact the patterns of health services and medicine utilisation in this population. Moreover, the participants of this study predominantly belong to larger cities of NZ. Therefore, these findings may not have captured the full spectrum of opinions of the Pakistani immigrants living in rural areas. Further research is required to investigate the challenges and perspectives of first‐generation Pakistani immigrants migrating to rural areas in NZ.

## 5 Conclusions

This study sheds light on the healthcare‐seeking behaviour, awareness, and experiences of Pakistani immigrants in NZ. The findings highlight a unique blend of traditional practices, self‐reliance, and adaptation to a new healthcare system. Participants primarily relied on home remedies and self‐medication for minor ailments, supplementing these approaches with conventional healthcare when necessary. Bringing medications from Pakistan underscores the challenges some immigrants face with accessibility and perceived adequacy of treatment options in NZ. The study revealed a general appreciation for the quality of care and professionalism within NZ's healthcare system, particularly its cautious approach to prescribing antibiotics and emphasis on comprehensive care. However, barriers such as cost, long waiting times, and limited knowledge of available pharmacy services were notable.

These insights underline the importance of culturally tailored healthcare strategies to address the expectations and challenges faced by immigrant populations. Strengthening awareness about accessible services, bridging cultural gaps, and reducing financial barriers could enhance the healthcare experience for this community.

## Author Contributions

**Noor A Mann:** formal analysis, writing–original draft, writing–review and editing. **Zain A Khan:** writing–original draft, writing–review and editing, formal analysis. **Saima Asghar:** data curation, investigation, formal analysis, writing–review and editing. **Afshan Rani:** investigation, methodology, data curation, writing–review and editing. **Nadia Hussain:** validation, writing–original draft, writing–review and editing. **Sumera S Akhtar:** data curation, formal analysis, writing–review and editing. **Susan Heydon:** supervision, conceptualisation, methodology, writing–review and editing. **Mudassir Anwar:** conceptualisation, methodology, supervision, writing–review and editing.

## Ethics Statement

The study received ethical approval from the University of Otago Human Ethics Committee (ref: D20/167). Before the interviews, eligible participants were briefed on the objectives of the study, and their verbal and written consent was acquired as an ethical prerequisite for their involvement.

## Conflicts of Interest

The authors declare no conflicts of interest.

## Supporting information

Supporting information.

## Data Availability

Data that support the findings of this study are available on request from the corresponding author. The data are not publicly available due to privacy or ethical restrictions.
