# Supplementary material for: Patterns of Health Services and Medicine Utilisation by First‐Generation Pakistani Immigrants in New Zealand
Source: Health Expect. 2025 Feb 2;28(1):e70169. doi: 10.1111/hex.70169 (PMC11788322; doi:10.1111/hex.70169)
Supplement: Supplementary file 1 — Supporting information. [file HEX-28-e70169-s001.docx]

**Interview guide:**

1. Demographic details such as age, educational background, how long in NZ, and occupation.
2. What’s your general impression of New Zealand health system?
3. What do you like about NZ health system?
4. What do you don’t like about NZ health system?
5. What difficulties/barriers you experience when accessing healthcare in New Zealand?
6. How did you gather information about New Zealand health system when you first came to New Zealand?
7. What health services in New Zealand are you aware of?

Prompt: The interviewer will then prompt about the services that the participant did not name and ask if they were aware of it?

1. What service(s) have you utilized?
2. What do you first do when you feel sick?
3. Do you do the same when your child is sick? If not, why?
4. How often do you visit a pharmacy when you feel sick?
5. How often do you visit a GP when you feel sick?
6. What characteristics of a GP are important to you when you visit them?
7. Are you aware of the services that community pharmacy can offer?

Prompt: Interviewer may then name the services one by one and ask if they were aware of them and if they have utilized that service?

1. Do you have any long-term condition(s)?
2. Do you have health insurance? If not, why?
3. Do you bring medicines from Pakistan? If yes, what type of medications and how you use them?
4. Do you use traditional/herbal/complementary medicines?
5. What type of illnesses you use traditional medicine and conventional medicine for?
